# A sepsis progression model in humans: characterization of biomarkers descriptive of sepsis progression

**DOI:** 10.1186/cc10406

**Published:** 2011-10-27

**Authors:** BW Footer, C-B Hsiao, DM Parish, P Wickremasingha, A-B Halim, G Senaldi, R Scheyer, JJ Schentag

**Affiliations:** 1 State University of New York at Buffalo, Buffalo, NY, USA; 2 CPL Associates, LLC, Buffalo, NY, USA; 3 Erie County Medical Center, Buffalo, NY, USA; 4 Daiichi Sankyo Pharma Development, Edison, NJ, USA

## Introduction

Previously our group has developed neural net progression models to characterize the development of organ failure in an ovine only as well as an integrated human/ovine model of acute lung injury using early clinical information. The goal of this study was to expand our model of disease progression using clinically available data as well as more exploratory biomarkers, such as the endotoxin activity assay (EAA), cytokines, D-dimer, copeptin, and procalcitonin, in an adult population with sepsis.

## Methods

Three North American study sites enrolled adult patients within 24 hours of meeting at least two SIRS criteria with clinical evidence of infection. Biomarker sampling occurred daily on days 1 to 7 and on days 14, 21, and 28. Clinical data from the 24 hours preceding the first sampling point as well as the baseline biomarker values were used as model inputs. Model outputs were serum creatinine (Scr) and organ metric (OM) over the study duration. OM is a composite parameter similar to the SOFA score with the CNS category removed and a continuous rather than categorical value. A neural net was used to perform a multiple parameter logistic regression while allowing for non-linear (usually sigmoidal) dependence on input parameters. Input parameters are first used individually to model the output and are then ranked based on the minimum mean squared error (MMSE) in these single-parameter models. The two parameters with the lowest MMSE are used to create the final multi-parametric model, which yields a lower modeling error than the original single-parameter models.

## Results

Thirty patients were enrolled with the two most common infection types being pneumonia and bloodstream. Seventy per cent of patients had at least one organ failure at enrollment. Diastolic blood pressure (DBP), red blood cell count (RBC), and copeptin had the smallest MMSE when individually predicting OM. Combining DBP and RBC yielded good agreement between the modeled and actual OM value (*r*^2 ^= 0.60). Individually, the prothrombin time (PT), copeptin, and phosphorus had the smallest MMSE when modeling Scr. The *r*^2 ^value between the model and actual Scr was 0.64 when combining PT and copeptin.

## Conclusion

When analyzed using a neural net model, changes in overall organ dysfunction and serum creatinine were predicted from early clinical data in a population of adult patients with sepsis. Identifying predictive biomarker patterns and coupling this information with known drug/intervention response could aid in optimizing treatment timing for greatest clinical benefit.

